# Genomic patterns in *Acropora cervicornis* show extensive population structure and variable genetic diversity

**DOI:** 10.1002/ece3.3184

**Published:** 2017-06-30

**Authors:** Crawford Drury, Stephanie Schopmeyer, Elizabeth Goergen, Erich Bartels, Ken Nedimyer, Meaghan Johnson, Kerry Maxwell, Victor Galvan, Carrie Manfrino, Diego Lirman

**Affiliations:** ^1^ Department of Marine Biology and Ecology Rosenstiel School of Marine and Atmospheric Science University of Miami Miami FL USA; ^2^ Department of Marine and Environmental Sciences Nova Southeastern University Dania Beach FL USA; ^3^ Mote Marine Tropical Research Laboratory Summerland Key FL USA; ^4^ Coral Restoration Foundation Key Largo FL USA; ^5^ The Nature Conservancy Summerland Key FL USA; ^6^ Federal Fish and Wildlife Conservation Commission Marathon FL USA; ^7^ Punta Cana Ecological Foundation Punta Cana Dominican Republic; ^8^ Central Caribbean Marine Institute Princeton NJ USA; ^9^ Little Cayman Research Centre Little Cayman Cayman Islands

**Keywords:** *Acropora cervicornis*, conservation genetics, ecological restoration, Florida Reef Tract, intraspecific diversity, Next Generation Sequencing, population genetics

## Abstract

Threatened Caribbean coral communities can benefit from high‐resolution genetic data used to inform management and conservation action. We use Genotyping by Sequencing (GBS) to investigate genetic patterns in the threatened coral, *Acropora cervicornis*, across the Florida Reef Tract (FRT) and the western Caribbean. Results show extensive population structure at regional scales and resolve previously unknown structure within the FRT. Different regions also exhibit up to threefold differences in genetic diversity (He), suggesting targeted management based on the goals and resources of each population is needed. Patterns of genetic diversity have a strong spatial component, and our results show Broward and the Lower Keys are among the most diverse populations in Florida. The genetic diversity of Caribbean staghorn coral is concentrated within populations and within individual reefs (AMOVA), highlighting the complex mosaic of population structure. This variance structure is similar over regional and local scales, which suggests that in situ nurseries are adequately capturing natural patterns of diversity, representing a resource that can replicate the average diversity of wild assemblages, serving to increase intraspecific diversity and potentially leading to improved biodiversity and ecosystem function. Results presented here can be translated into specific goals for the recovery of *A. cervicornis*, including active focus on low diversity areas, protection of high diversity and connectivity, and practical thresholds for responsible restoration.

## 1 INTRODUCTION

Coral reefs are among the most biodiverse and productive ecosystems on earth but are declining due to a combination of natural and anthropogenic stressors (Hughes et al., 2003; Pandolfi et al., 2003). In the Caribbean, declines have been particularly dramatic (Gardner, Cote, Gill, Grant, & Watkinson, 2003). As efforts to remedy reef deterioration continue, high‐quality genomic data are useful to help understand population structure, connectivity, diversity, and the adaptive potential of corals (van Oppen & Gates, 2006; van Oppen, Oliver, Putnam, & Gates, 2015) that form the structural basis needed to sustain reef biodiversity and function. These data are informative from several perspectives: (1) genetic diversity is related to resilience and resistance to ongoing stress, (2) genetic diversity influences interspecific diversity and ecosystem function, providing important information on reef health, and (3) coral reef restoration and management require better information about factors that dictate the spatial scale of focal areas, as well as a strong conceptual understanding of the consequences of active intervention on individual reefs.

*Acropora cervicornis*, listed as threatened under the US Endangered Species Act (Hogarth, 2006), has declined by more than 95% in some parts of the Caribbean (Miller, Bourque, & Bohnsack, 2002). This species is a major reef builder with high growth rates (Tunnicliffe, 1981) and frequent asexual reproduction via fragmentation (Highsmith, 1982) that binds sediments and creates essential structure for associated reef organisms. These characteristics make it ideally suited for restoration using the coral gardening technique (Epstein, Bak, & Rinkevich, 2001; Rinkevich, 2005). Given *A. cervicornis’* prominent role in reef function and potential for population modification due to active intervention, patterns of genetic diversity represent a fundamental connection between ecological, evolutionary, and management processes (Vellend & Geber, 2005). A major goal of most conservation plans is to conserve species biodiversity, but a focus on intraspecific genetic variation, the most basic source of biodiversity (Hughes, Inouye, Johnson, Underwood, & Vellend, 2008), should be a focal point of conservation efforts motivated to build evolutionary resilience (Sgro, Lowe, & Hoffmann, 2011).

Intraspecific diversity can predict community structure (Crutsinger et al., 2006) and has important consequences at the ecosystem level (Forsman & Wennersten, 2015), especially in foundation or dominant species (Barbour et al., 2009; Whitham et al., 2003). Genetic diversity also increases resistance and resilience when coping with disturbance (Forsman & Wennersten, 2015; Hughes & Stachowicz, 2004; Jahnke, Olsen, & Procaccini, 2015; Jump, Marchant, & Peñuelas, 2009; Reusch, Ehlers, Hammerli, & Worm, 2005). High standing genetic variation can allow organisms to adapt to changing environments (Oliver et al., 2015) and form communities in which variation may still exist after disturbances occur (Jahnke et al., 2015), increasing overall resilience (Reusch et al., 2005). Although neutral genetic markers, like those investigated here, are not expected to have ecological consequences per se, they represent diversity which may be functionally significant and relevant for management. These patterns can be used to guide conservation actions aiming to develop evolutionary resistance, or the ability to cope with changing conditions, by increasing genetic variability and maintaining adaptive potential (Oliver et al., 2015; Sgro et al., 2011). This goal requires a solid understanding of focal species’ population structure and diversity.

Understanding genetic patterns in *A. cervicornis* is especially important because active restoration has emerged as one method for mitigating ongoing declines of this species in the Caribbean (Lirman et al., 2010). Along the FRT, a network of seven coral nurseries operated by six organizations (Coral Restoration Foundation, Florida Fish and Wildlife Conservation Commission, Nova University, Mote Marine Lab, The Nature Conservancy, and University of Miami) is responsible for *A. cervicornis* restoration (Lirman et al., 2014). These nursery populations are ecologically and evolutionarily important because they represent the functional unit (i.e., source of coral used in restoration) of the active conservation response to declining reefs in Florida.

Early investigations of *A. cervicornis* found many colonies of single or few genotypes within reefs using a self‐recognition assay, where clonality was considered common at the scale of 10 m (Neigel & Avise, 1983). An early population genetics study found reefs as close as 2 km showed fine‐scaled genetic differentiation, but this was partly due to introgression signatures from *Acropora palmata* at a single reef (Vollmer & Palumbi, 2007). At regional scales of >500 km, significant population structure was common (Vollmer & Palumbi, 2007). These results demonstrated the importance of local recruitment, showing the need for regionally focused management strategies based on the populations and demographics of each region (Vollmer & Palumbi, 2007).

Studies in Florida found similar trends of regional‐scale population across the Caribbean, but high connectivity within the FRT (Baums, Johnson, Devlin‐Durante, & Miller, 2010; Hemond & Vollmer, 2010). Florida also had relatively high genetic diversity, meaning drastic population declines (Miller et al., 2002) have not disproportionately affected the genetic composition of corals in this region. Population structure was also found at scales less than 5 km in Puerto Rico (Reyes & Schizas, 2010) and on the Meso‐American Barrier Reef in *A. palmata* (Porto‐Hannes et al., 2015). Drury et al. (2016) described population structure within the Florida Reef Tract and between Florida and the Dominican Republic and supported previous findings of diversity localized within populations.

We use Genotyping by Sequencing (GBS; Elshire et al., 2011), a method which takes advantage of reduced genome complexity produced by restriction enzymes, to produce large numbers of Single Nucleotide Polymorphisms (SNPs). These markers, which are distributed throughout the genome, can be used to analyze within‐species diversity, infer demography, understand evolutionary processes, and uncover cryptic genetic variation (Gibson & Dworkin, 2004; Luikart, England, Tallmon, Jordan, & Taberlet, 2003). These data can contribute to a better understanding of genetic diversity within reefs, facilitating responsible restoration (Baums, 2008). We take advantage of individual wild reefs and previously collected nursery corals representing large regions to produce a hierarchical sampling methodology that utilizes corals from the entire Florida Reef Tract, including within regions and within reefs, to examine *A. cervicornis* genetic diversity over multiple spatial scales relevant to conservation and management actions. We also analyze samples from the Cayman Islands (Central Caribbean Marine Institute), Dominican Republic (Punta Cana Ecological Foundation), and Belize (Fragments of Hope) to contribute to the growing knowledge of Caribbean‐wide population structure for this species (Baums, Miller, & Hellberg, 2005; Drury et al., 2016; Hemond & Vollmer, 2010; Porto‐Hannes et al., 2015; Reyes & Schizas, 2010; Vollmer & Palumbi, 2007). We test the hypotheses that (1) there is population structure within the Florida Reef Tract and western Caribbean, (2) collections from in situ nurseries represent high genetic diversity, (3) diversity among individuals from nursery stocks is comparable to genetic variation of colonies on wild reefs, and (4) genetic diversity is uneven across the span of the FRT. Data presented here form a concrete link between analysis of population genetics and management efforts.

## 2 MATERIALS AND METHODS

### 2.1 Sample collection

**Figure 1:**
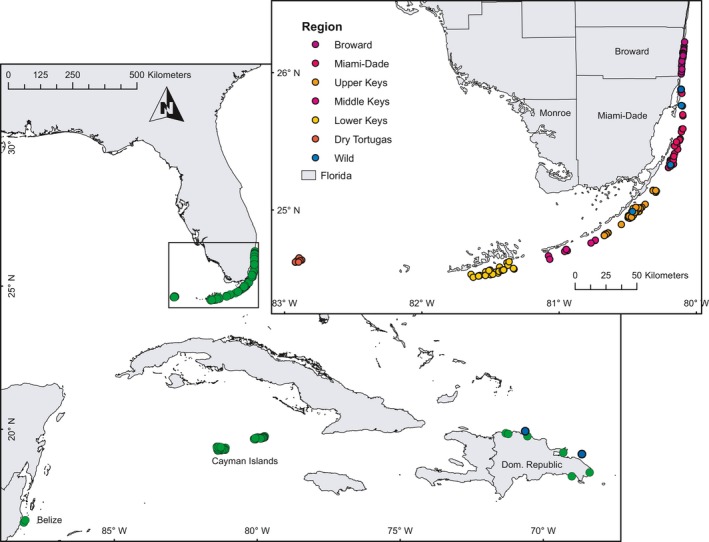
Map of collection locations. Large panel shows regional collections from Belize, the Cayman Islands, the Dominican Republic, and the Florida Reef Tract (FRT). Inset shows locations of Florida collections, color coded by regional population. “Wild” collections within Florida (*n* = 4) and the Dominican Republic (*n* = 2) are locations where multiple samples were collected from individual reefs, represented by blue dots

Samples for this study were collected between June 2012 and August 2015 from (1) nurseries representing regional populations and (2) individual wild reefs. Nursery stocks are composed of single colonies originally collected from individual reefs spanning the study area (typically one colony per reef) with the goal of minimizing collection of clonemates and maximizing genotypic and genetic diversity. Rarely, several samples in a nursery were collected from the same individual reef, but were presumed to represent nonclonemates due to distance separating colonies and sequencing results of this study. These nurseries contained *A. cervicornis* colonies from along the Florida Reef Tract, Dominican Republic, Cayman Islands, and Belize and represent a broad spatial sampling strategy (Figure 1). For comparison, wild collections were made during 2015 from 4 to 20 haphazardly selected discrete colonies on each of six individual reefs (Table 1; Figure 1 blue points) in Florida (*n* = 4) and the Dominican Republic (*n* = 2). These individual wild reef samples represent a much higher‐density sampling method. For each colony, approximately 0.5 cm apical tips were collected with a razor blade, transferred to 250 μL of chaotropic salt preservative (“Chaos,” 4.5 mol/L guanidinium thiocynate, 2% N‐lauroylsarcosine, 50 mmol/L EDTA, 25 mmol/L Tris–HCL pH 7.5, 0.2% antifoam, 0.1 mol/L 2‐mercaptoethanol) and stored at 4°C until processing. Overall, samples were collected from 177 individuals across entire Florida Reef Tract (Figure 1), with additional colonies from the Cayman Islands (*n* = 53), Dominican Republic (*n* = 29), and Belize (*n* = 6). In addition, 67 samples from four wild reefs in Florida and two wild reefs in the Dominican Republic (Table 1) were collected for comparisons of nursery and wild reef diversity.

**Table 1:** Sample collection locations

Location	Population	N colonies “Regional”	Wild reef	N Colonies “Wild”
Florida	Broward	24		–
	Miami‐Dade	48	Miami	20
			Cheetos	13
			Sunny Isles	4
	Upper Keys	48	Tavernier	15
	Middle Keys	17		–
	Lower Keys	31		–
	Dry Tortugas	9		–
Belize		6		–
Cayman Islands		53		
Dominican Republic		29	Cayo Carenero	7
			Punta Rusia	8
Total N		265		67
N Loci Analyzed		3,136		3,165

Number of samples processed from each population. Regional populations were collected from nurseries (see Methods) except for the Cayman Islands, which was sampled using the same protocol but was not included within a nursery. Wild samples were collected from four reefs in Florida (“Cheetos,” “Miami,” “Sunny Isles” in Miami‐Dade, “Tavernier” in the Upper Keys) and two reefs in the Dominican Republic (“Punta Rusia” and “Cayo Carenero”).

### 2.2 DNA isolation and library preparation

Samples (skeleton and preservative) were homogenized using silica beads in original collection tubes, and DNA was extracted using a silica column protocol and a vacuum manifold modified from Ivanova, Dewaard, and Hebert (2006). DNA degradation was evaluated using gel electrophoresis. Extracted DNA was quantified in triplicate (AccuBlue™ High‐Sensitivity dsDNA Quantitative Solution), and 100 ng of DNA from each sample was dried down in 96‐well plates. Libraries were prepared using a modified protocol of Elshire et al. (2011). Briefly, samples were hydrated in 5 μL water and digested with ApeKI. ApeKI is a partially methylation‐sensitive restriction enzyme, which reduces cut sites in repetitive regions of the genome and enhances cuts in lower‐copy regions (Elshire et al., 2011). After digestion, SPRI bead size‐selection was used to eliminate small fragments (<100 bp); 4‐ to 8‐bp barcodes unique to each sample and a common adapter were ligated to fragments (see Elshire et al. 2011 for adapter sequences). Ligated samples were pooled and bead purified to select fragments in the 100–250 bp size range. Pooled samples were amplified using PCR with primers complementary to the adapters, which also produces an overhang complimentary to the oligonucleotides used in Illumina flow cells for sequencing. PCR products were bead purified, eluted in 10 mmol/L Tris, and fragment size distribution was analyzed on an Agilent Bioanalyzer. Samples were sequenced using single‐end 75‐bp reads on an Illumina HiSeq 2500 (Elim Biopharmaceuticals Inc., Hayward, CA, USA).

### 2.3 Data processing

Raw sequences were processed using a parsing script modified from Melo, Bartaula, and Hale (2016) to remove reads without barcode and cut sites. These reads were trimmed using Trimmomatic 0.32 (Bolger, Lohse, & Usadel, 2014) to remove low quality bases at the leading and trailing end and remove reads if a 4‐bp sliding window average read quality fell below 20. Reads were demultiplexed according to barcode and aligned to the *Acropora digitifera* genome (Shinzato et al., 2011) using Bowtie2 (Langmead, Trapnell, Pop, & Salzberg, 2009). Aligned reads were processed using GATK 3.4.2 (McKenna et al., 2010) following a protocol modified from existing best practices to produce the SNP dataset (Auwera et al., 2013; DePristo et al., 2011). Briefly, optical/sequencing duplicates were marked with MarkDuplicates, and HaplotypeCaller was used to produce initial genotypes under the cohort analysis framework. Base Quality Score Recalibration was conducted, but due to multiplexing, some samples had too few bases within read groups for adequate model convergence. Intermediate gVCF files were merged and genotyped using Genotype‐gVCFs. This initial SNP matrix was recalibrated with known variants, which were bootstrapped from replicate samples. Six pairs of biological replicates (from samples not analyzed in this dataset) with the highest number of reads were used to find “known sites” using filtering through TASSEL 5.2.29 (Bradbury et al., 2007) to detect loci that were consistently genotyped in replicate pairs. Matching calls between replicates were then used as “known sites” to train Variant Quality Score Recalibration, using the annotation values of known sites to determine thresholds for accepting SNPs. Tranche plots were examined, and a truth sensitivity of 99.9 was used for recalibration, a permissive setting which favors discovery of novel variants.

Called SNPs were quality‐filtered following Drury et al. (2016) to produce two datasets for comparison, (1) regional populations collected from nurseries and (2) wild populations collected from individual reefs. Separate datasets were used because independent assessment of each subset of samples allowed optimization of number of loci per sample, allelic patterns, and independent AMOVAs. Briefly, the SNP matrix was filtered iteratively using TASSEL 5.2.29 (Bradbury et al., 2007) to select loci called in at least 50% of individuals and individuals with at least 30% of loci called. LinkImpute (Money et al., 2015) was used to impute missing genotypes based on the 10 nearest neighbors, suitable for calling inbred (low He) samples typical of the coral patterns found here. The final SNP matrix included loci in >50% of individuals and individuals with >45% of loci and did not substantially change the described patterns from nonimputed data. To limit the analysis to neutral loci, loci with excessive heterozygosity out of Hardy–Weinberg Equilibrium (*p* < .01) were identified using Arlequin 3.5.1 (Excoffier & Lischer, 2010) with 1,000,000 steps and removed. Next, to remove loci that were not independently associated (i.e., in linkage equilibrium), linkage disequilibrium (LD) was calculated using TASSEL with a 50 SNP sliding window and loci were removed (*R* ^2^ > 0.2 or *p* < .01). LOSITAN (Antao, Lopes, Lopes, Beja‐Pereira, & Luikart, 2008) was used with forced neutral mean *F*_ST_ and 50,000 simulations to find non‐neutral outlier loci (locus‐specific *F*_ST_ *p* < .01), which were removed. Finally, clonality was assessed using pairwise genetic distance of eight samples collected from a single colony, calculated in TASSEL using the formula of Endelman and Jannink (2012). Samples from the same site below the arbitrary 66th‐% threshold (0.075) were removed (*n* = 8).

### 2.4 Analyses

Population differentiation, including AMOVA (Excoffier, Smouse, & Quattro, 1992) and pairwise *F*_ST_ was analyzed in Arlequin (3.5.1), with 10,000 permutations for significance values. Discriminant Analysis of Principal Components (DAPC) was completed using the *adegenet* package in R (Jombart, 2008) to visualize differentiation, with populations defined as in Table 1. Molecular diversity indices and allelic patterns were analyzed in GenAlEx 6.502 (Peakall & Smouse, 2006). Isolation by distance (Mantel's test) was evaluated within Florida using the package *ade4* (Dray & Dufour, 2007) with matrices of genetic distance between each sample (calculated in TASSEL using formula of Endelman and Jannink 2012) and geographic distance between each sample (calculated in GenAlEx 6.502), using 9,999 permutations. Genetic diversity within the Florida Reef Tract was visualized with ArcGIS toolbox *Genetic Landscapes GIS Toolbox* 10.1.3 (Vandergast, Perry, Lugo, & Hathaway, 2011) plotting genetic distance between points (calculated in TASSEL) and the nearest 30 samples and producing an IDW interpolation. Comparison of genetic diversity based on sample size was completed by creating random subsets of individuals from the Miami‐Dade population. Samples (*n* = 2–25 individuals) were taken from the population 50 times with replacement, and *adegenet* (Jombart, 2008) was used to calculate expected heterozygosity for each subsample.

## 3 RESULTS

### 3.1 Interpopulation differentiation

In total, 265 samples from regional populations and 67 samples from wild reefs were analyzed at 3,136 and 3,165 loci, respectively (Table 1). In the regional pairwise comparisons, significant *F*_ST_ values occurred in about 70% (25 of 37) of comparisons (Table 2). Within Florida, average pairwise *F*_ST_ values were between 0.033 and 0.059, although the Dry Tortugas population was not significantly different from any other population on the FRT. All Caribbean populations (Cayman Islands, Belize, Dominican Republic) were significantly different from each other but show variable relationships with populations in Florida. *F*_ST_ values ranged from 0.012 to 0.171, including four comparisons between Belize and other populations with *F*_ST_ values greater than 0.1.

**Table 2:**
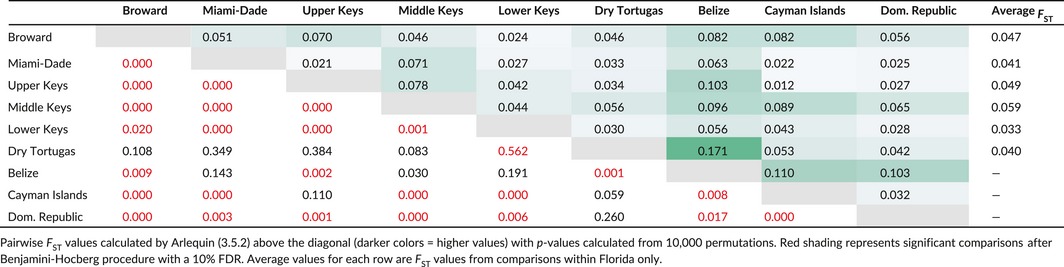
Pairwise *F*_ST_ values between populations

**Figure 2:**
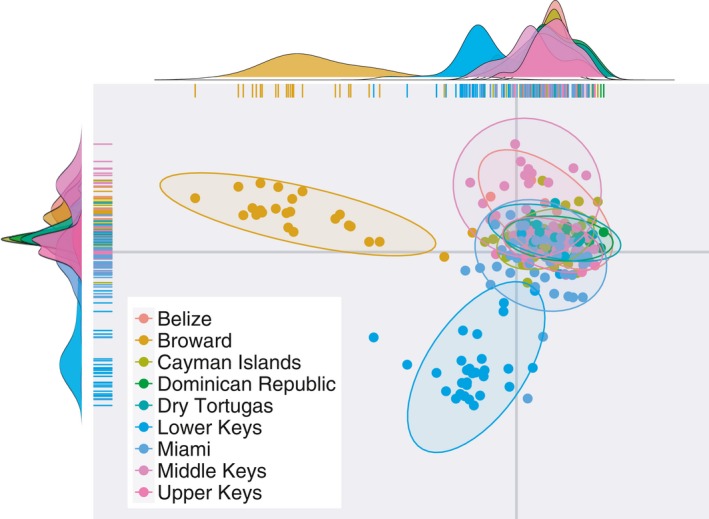
Discriminant analysis of principal components for all populations. Colors assigned by population for Broward, Miami‐Dade, Upper Keys, Middle Keys, Lower Keys, Dry Tortugas, Belize, Cayman Islands, and Dominican Republic. Top density plot represents Discriminant Function 1, and side density plot represents Discriminant Function 2

**Figure 3:**
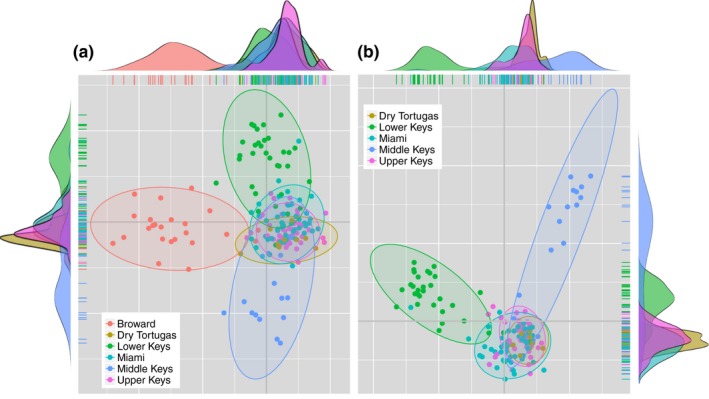
DAPC for (a) Florida Populations and (b) Florida Populations without Broward. Colors assigned in both plots by population for Broward, Miami‐Dade, Upper Keys, Middle Keys, Lower Keys, Dry Tortugas. Top density plot represents Discriminant Function 1, and side density plot represents Discriminant Function 2

Discriminant Analysis of Principal Components showed variable population separation, with Broward and Lower Keys separating from the core cluster in the regional analysis (Figure 2). Overlap between Miami‐Dade, Upper Keys, Dry Tortugas, Cayman Islands, and Dominican Republic supported similarities based on pairwise *F*_ST_ values (Miami‐Dade—Dry Tortugas, Caymans—Upper Keys, etc.) although patterns of structure in the *F*_ST_ comparisons between Broward and other Florida populations are not apparent. Among Florida populations, the same outliers were evident, along with strong overlap between Upper Keys, Miami Dade, and Dry Tortugas (Figure 3a). When Broward populations are removed for visualization, the Middle Keys population showed strong differentiation, corresponding to large *F*_ST_ values.

For regional samples, an AMOVA was structured with Florida, Dominican Republic, Cayman Islands, and Belize as groups, with populations assigned within Florida. Nearly all genetic variation was within populations, with smaller fractions among groups and among populations within groups (Table 3a). A separate AMOVA on wild samples was structured with Florida and the Dominican Republic as groups, with wild reefs treated as populations within each (four in Florida two in the Dominican Republic). Variation was largely within populations, but individual reefs showed much higher variation among groups and among populations within groups (Table 3b) than the regional AMOVA (Table 3a). The AMOVA for wild samples also showed relatively high percent variation among populations within groups.

**Table 3:** Analysis of molecular variance

Source	*df*	SS	Variance	Percent variation
(a) Regional AMOVA				
Among groups	3	183.54	−0.42	−1.7
Among populations within groups	5	484.69	1.29	5.23
Within populations	523	12,497.86	23.90	96.47
(b) Wild AMOVA				
Among groups	1	157.29	1.41	4.41
Among populations within groups	5	518.07	3.50	10.97
Within populations	147	3,965.95	26.97	84.62

AMOVA for (a) regional populations and (b) wild populations. Groups in regional analysis were Florida, Cayman Islands, Dominican Republic, and Belize, with populations assigned within Florida as in Table 1 (by Population). Groups in wild analysis were Florida and the Dominican Republic, with four populations assigned within Florida and two populations assigned within the Dominican Republic as in Table 1 (by Wild Reef).

### 3.2 Intrapopulation differentiation

The nine regional populations examined had between six and 53 samples per population, although all except Dry Tortugas and Belize had at least 17 samples. Average allelic richness ranged from 1.023 to 1.247 for all populations. Observed heterozygosity was much lower than expected for all populations. Locally common alleles (frequency >5% within a population, but frequency <5% in 75% or more of populations) vary nearly tenfold from about 0.004 (Dry Tortugas and Belize) to 0.036 (Lower Keys). These alleles may be disproportionately influential and explain some of the spread in DAPC, where higher values correspond to some outlying clusters (i.e., Broward, Lower Keys, but see Belize and Miami‐Dade) (Figure 2).

### 3.3 Geographic patterns of genetic differentiation

**Figure 4:**
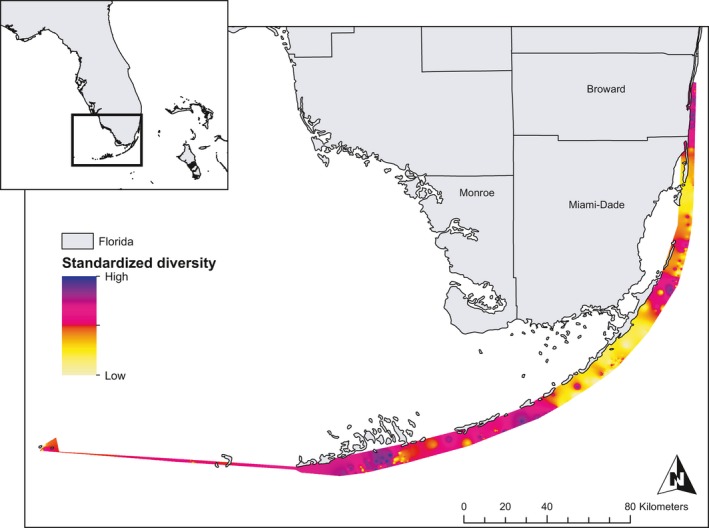
Interpolation of Genetic Diversity Across the Florida Reef Tract. Values are interpolations using the nearest 30 neighbors' pairwise genetic distance values as a diversity metric. Interpolation is on a standardized scale (0–1) and represents relative differences in genetic diversity

There is significant Isolation by Distance within Florida (Mantel Test, Correlation = 0.09, *p* = .012). The Lower Keys, Middle Keys, and Broward regions show the highest diversity values, while the Upper Keys and the ecotonal habitats between Broward and Miami‐Dade counties show the lowest diversity (Figure 4). The “safety valve” of Biscayne Bay, an area where the tidal flow of water from Biscayne Bay disrupts temperature and salinity on adjacent reefs, and areas surrounding the port of Miami may influence connectivity between Miami‐Dade and Broward, leading to small‐scale (~10 km) depression of diversity between Miami‐Dade and Broward populations. Sampling locations (Figure 1) covered most of the distribution of *A. cervicornis* in Florida, although gaps between the Marquesas and Key West (Lower Keys), the Upper Keys and Miami‐Dade, and Palm Beach County have not been extensively sampled.

### 3.4 Comparing regional nursery populations to individual reefs

**Figure 5:**
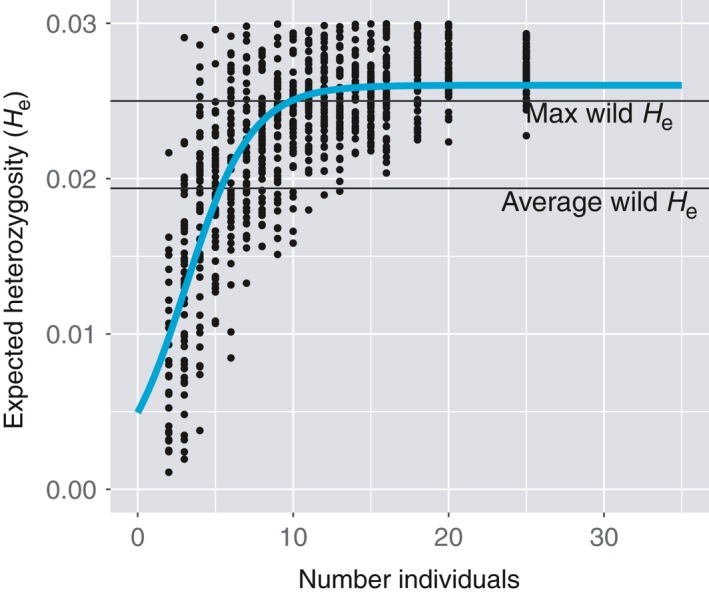
Expected heterozygosity as a function of sample size. Miami‐Dade regional population (*n* = 48) was subsampled randomly 50 times for each number of individuals. Mean wild expected heterozygosity and maximum wild expected heterozygosity are black reference lines. Blue line is logarithmic best fit

Regional nursery populations in Florida have similar or larger values of genetic diversity than most individual wild reefs (Table 4). Average allelic richness and expected heterozygosity are higher in nursery populations than on wild reefs, although the values are similar and show logarithmic growth as sample size increases (Figure 5; Fig. S1). Predicted asymptotes (i.e., maximum) expected heterozygosity for wild reefs, and regional nursery populations were 0.025 and 0.026, respectively (Fig. S1). Using expected heterozygosity as a diversity metric, approximately five individuals need to be collected and propagated within each regional nursery to meet average heterozygosity of wild reefs (Figure 5). To equal maximum expected heterozygosity from single wild reefs, approximately 10 individuals need to be collected and maintained within regional nursery populations. As the number of individuals maintained within nurseries increases beyond 10, no substantial increase in genetic diversity occurs (Figure 5).

**Table 4:** Patterns of genetic diversity in regional and wild reef populations

	Population	*n*	Na	Na Freq > 5%	nPrivate	nLocal	He	Ho	Polymorphism (%)	Na‐1/N	He/N
Regional	Broward	24	1.180	1.088	0.041	0.027	0.024	0.002	18.2	1.139	0.00101
	Miami‐Dade	48	1.247	1.043	0.081	0.026	0.018	0.002	24.7	1.226	0.00039
	Upper Keys	48	1.174	1.025	0.062	0.015	0.013	0.001	17.4	1.154	0.00027
	Middle Keys	17	1.142	1.134	0.042	0.024	0.024	0.002	14.2	1.083	0.00143
	Lower Keys	31	1.240	1.092	0.070	0.036	0.026	0.003	24.0	1.208	0.00085
	Dry Tortugas	9	1.034	1.034	0.014	0.004	0.011	0.001	4.6	0.923	0.00125
	Belize	6	1.023	1.023	0.005	0.004	0.009	0.001	2.6	0.856	0.00148
	Cayman Islands	53	1.128	1.029	0.042	0.011	0.011	0.001	12.8	1.109	0.00020
	Dominican Republic	29	1.128	1.071	0.042	0.013	0.015	0.001	12.8	1.094	0.00053
Wild	Cayo Carenero	7	1.043	1.043	0.010	0.000	0.014	0.002	4.30	0.900	0.00201
	Punta Rusia	8	1.033	1.033	0.005	0.000	0.010	0.002	3.35	0.908	0.00127
	Cheetos	13	1.067	1.060	0.016	0.000	0.014	0.003	6.70	0.990	0.00111
	Miami	20	1.115	1.097	0.048	0.000	0.018	0.004	11.53	1.065	0.00092
	Sunny Isles	4	1.014	1.014	0.000	0.000	0.006	0.002	1.42	0.764	0.00142
	Tavernier	15	1.097	1.084	0.029	0.000	0.017	0.003	9.67	1.030	0.00112

*n *= number of samples, Na = allelic richness, NaFreq > 5 = allelic richness greater than frequency 5%, nPrivate = percentage private alleles, nLocal = percentage locally common alleles (greater than 5% frequency within population but <5% in at least 75% of populations), He = expected heterozygosity, Ho = observed heterozygosity, Polymorphic loci = percentage of loci that are polymorphic, Na‐1/*n* = allelic richness by sample, He/N = expected heterozygosity by sample.

Locally common alleles are nonexistent in wild populations in this dataset, although this is likely a result of the low number of populations examined, low sample size, and the influence of geographic distance between the two groups (Florida and Dominican Republic). Although percentage of variation for Among Group and Among Population was larger in the Wild AMOVA than Regional, similar patterns of variation were maintained. Approximately 84% (Table 3b) of variation in wild reefs is within individual reefs. This is less than in the regional populations (96%, Table 3a) but much larger than the other sources of variation. As regional nursery samples were collected over broader geographic ranges, this supports Isolation by Distance shown in this species, because “groups” as defined in AMOVA cover different spatial scales in the two datasets. Allelic richness tracks closely between wild populations and nursery groups as sample size increases, although exhaustive sampling of individual reefs may lead to higher diversity values (Fig. S1). In addition, patterns of population structure (Table S1) and DAPC for wild populations (Fig. S2) are similar to regional populations.

## 4 DISCUSSION

Increasing genetic resolution has shown that population structure and genetic patchiness occurs over progressively smaller scales in *A. cervicornis* (Drury et al., 2016; Vollmer & Palumbi, 2007). In this study, we expand on this work to resolve previously unknown population structure within the Florida Reef Tract and between more distant populations in the Caribbean. Information on these patterns is useful for management and conservation, especially in species where active intervention is ongoing.

*Acropora cervicornis* populations in Florida show a mosaic of diversity and extensive population structure, where about 2/3 of pairwise *F*_ST_ comparisons were significant and most genetic variation occurs within populations. These results support general population differentiation found within the FRT, but contradict some previously described specific pairwise comparisons (i.e., Miami‐Dade and the Lower Keys) (Drury et al., 2016). The Dry Tortugas population showed no significant pairwise *F*_ST_ values, although the average *F*_ST_ (0.040) is comparable to other Florida populations. This relationship is like due to the small sample size, but may also relate to the upstream location of this population in the Florida Current (Schmitz & Richardson, 1991). Importantly, the Dry Tortugas represents the nursery with the fewest “genotypes” along the FRT, so small sample size in this study is representative of patterns in restoration resources, if not of larger natural populations.

The Cayman Islands and Dominican Republic represent populations that were previously under‐ or unexamined with genetic or genomic techniques, resolving population structure typical of longer distances in Caribbean Acroporids (Baums, Miller et al., 2005, 2010; Hemond & Vollmer, 2010; Vollmer & Palumbi, 2007). All samples examined here fall west of the Mona Passage, considered a barrier to connectivity between the Eastern and Western Caribbean (Baums, Miller et al., 2005; Taylor & Hellberg, 2006); however, connectivity within the western area of the Caribbean does not preclude finer‐scale population structure, and pairwise comparisons between these populations exhibit a range of differentiation. The Cayman Islands were significantly different from all Florida populations except the Upper Keys and Dry Tortugas, highlighting the mosaic nature of population structure. Similarly, Belize shows highly variable population structure when compared to Florida, with *F*_ST_ values varying threefold, although these results must be interpreted with extreme caution due to limited sampling. Caribbean‐wide patterns of structure exhibited in this dataset highlight the complexity of long‐distance relationships and the fine‐scale structure of populations within Florida. Population structure may also be influenced by the presence of unique alleles that are disproportionately influential in DAPC, such as those in Broward, which separates from all other populations in the first discriminant function. This collapse of variation is not shown in pairwise *F*_ST_ calculations because all loci were used to examine structure, rather than those that drive the majority of variation. The Middle Keys area is also uniquely isolated, with the highest average *F*_ST_ values and significant population structure between every other Florida population except the Dry Tortugas, which is supported by the Within‐Florida DAPC. These patterns may be related to environmental conditions across the FRT, including the influx of high‐salinity, high‐temperature inimical waters from Florida Bay across the reefs in the Middle Keys (Lirman & Fong, 2007).

The allelic patterns resolved here show that Florida populations fall in the same range as the Cayman Islands and Dominican Republic, indicating that this region is not genetically depauperate and may not have suffered disproportionate loss of genetic diversity during population declines, corresponding to earlier findings (Hemond & Vollmer, 2010). This result is tentative, however, as simulated population declines require generations to pass before observable genetic diversity declines (Tajima, 1989). Although the FRT has generally elevated genetic diversity, it is uneven and values for expected heterozygosity are highest in Broward, the Middle and Lower Keys, corresponding to outlier groups in DAPC, which may be important resources as repositories of standing variation (Schopmeyer et al., 2012). Allelic patterns also illustrate areas of high diversity existing over small scales, for example, The Lower Keys and Miami‐Dade have higher diversity than the entire Cayman Islands, despite differences in scale. Expected Heterozygosity and Allelic Richness scale with sample size, so results from Belize and the Dry Tortugas must be viewed with caution. However, expected heterozygosity is asymptotic beyond approximately 10–15 samples (Figure 4), so variation in sample size above this threshold is unlikely to mask natural patterns.

Observed heterozygosity is much lower than expected for all populations, which may be an effect of biological or technical influences. Potential inbreeding within isolated *A. cervicornis* populations supports increased genetic diversity as a management objective (Sgro et al., 2011), but partitioning of subpopulations not recognized in this analysis may also contribute to this effect, where different allele frequencies in separate areas can lead to calculations of high expected heterozygosity (Wahlund effect). Observed heterozygosity values are relatively consistent across regions, but are much lower than values found by sequencing individual genes (Hemond & Vollmer, 2010; Vollmer & Palumbi, 2007), which may be partially due to the biallelic nature of data used in this analysis (Elshire et al., 2011). Given these differences, absolute values of specific metrics should not be compared between techniques. These results may also be related to pipeline artifacts (minor allele frequency and ratio thresholds) and the use of a Pacific congeneric as reference (*A. digitifera*), where nonreference alleles are assumed to be errors unless sufficient evidence in the dataset suggests otherwise. Due to multiplexing in the laboratory protocol, reads per individual were limited, which could lead to inflated homozygote calls as the secondary allele was missed, contributing to an underestimation of heterozygotes during read processing. Individuals with low heterozygosity did not disproportionately influence the results; however, this is an important potential bias in most restriction‐digest sequencing that would affect all populations within the study.

As restoration efforts mature and expand throughout the Caribbean (Young, Schopmeyer, & Lirman, 2012), incorporating genetic data in management and restoration becomes increasingly important (Baums, 2008; Drury & Lirman, 2017; Lirman & Schopmeyer, 2016) for creating natural assemblages that will be resistant, resilient, and maintain adaptive potential. The capability to replicate the genetic patterns on wild reefs is an important component of this process. The AMOVA for each dataset shows similar patterns of concentrated genetic variation localized within populations and potential residual subpopulation structure “Among populations within regions.” Allelic patterns also show that nursery populations (regions) harbor genetic diversity (He and Na) slightly elevated with respect to individual reefs sampled in Florida and the Dominican Republic, suggesting restoration networks contain appropriate, representative genetic resources. Expected heterozygosity and allelic richness closely track as sample size increases between both groups (Fig. S1), so using more nursery‐based individuals can create more diverse outplanting assemblages. On average, five individuals are needed from a regional nursery population to reach mean diversity values for an individual reef during restoration. Genetic diversity of an assemblage made of individual corals varies depending on the genetic differentiation between individuals, so while five individuals meet the mean expected heterozygosity of a wild reef, at least 10 are needed to ensure that sampling effects meet this limit, that is, all randomly selected assemblages fall above the threshold. Ten individuals in an assemblage also replicates, on average, the maximum expected heterozygosity from a wild reef in this study, meaning this figure is an important target for management and conservation. The variability shown by subsampling corals from the nursery also illustrates how genotypes harbored in nurseries can be more or less distantly related, influencing the diversity of outplanted assemblages and suggesting that the use of nonclonemates, regardless of mutlilocus genotype, can be used to achieve genetic variation and adaptive potential. Although genetic diversity increases marginally beyond 10 samples, genotypic diversity is also very important. Reproductive compatibility (Baums, Hughes, & Hellberg, 2005; Fogarty, Vollmer, & Levitan, 2012), unforeseen growth, and survivorship patterns mediated by genotype by environment interactions (Drury, Manzello, & Lirman, 2017) and genotype x genotype interactions with *Symbiodinium* (Baums, Devlin‐Durante, & LaJeunesse, 2014) are also important components of the diversity of an assemblage which make diverse, resilient reefs. One limitation of this assessment is that population declines have likely decreased genetic diversity on individual reefs to some degree, leading to current estimates from wild reefs that are not reflective of historical healthy reefs. Using changes in sample size (Fig. S1) and modeling differences in diversity within nursery populations, some concern about whether small sample size accurately reflects diversity of larger assemblages can be tempered because asymptotes for expected heterozygosity are similar for wild (0.025) and regional (0.026) populations. However, if population declines removed large portions of genetic variation related to stress tolerance (i.e., nonhearty individuals harbored large amounts of genetic variation), current values may not be representative. The individual patch reefs in this study remain among the healthiest populations of *A. cervicornis* in Florida today, so estimates made here reflect the best current data on intraspecific diversity on healthy reefs.

While there is significant isolation by distance in this dataset, small‐scale chaotic patchiness is evident through pairwise population structure in Florida and the wider Caribbean, mapping standardized diversity, the presence of variation among populations in AMOVA, and the presence of private and locally common alleles. This result is common of marine organisms despite the expectation that variation (genomic, transcriptomic, or phenotypic) would be homogenized over small scales by gene flow (Barshis et al., 2013; Drury et al., 2017; Hedgecock & Pudovkin, 2011; Kenkel, Almanza, & Matz, 2015; Kenkel & Matz, 2016; Warner, Oppen, & Willis, 2015) and may be due to a combination of factors including differential selection, environmental heterogeneity, differential mortality during long‐term population declines, or random effects producing large variation in recruitment success (i.e., Sweepstakes Reproductive Success) (Eldon, Riquet, Yearsley, Jollivet, & Broquet, 2016; Hedgecock & Pudovkin, 2011). Nonselective effects may also reconcile population structure with the apparent lack of local adaptation in this species (Drury et al., 2016). In *A. cervicornis,* these patterns are complex to resolve because of the influence of asexual reproduction, where demographic effects preceding population decline are now interacting with potentially poorly connected populations due to the Allee effect. In the comparisons of wild reefs, the “Within‐Population” source of variance represents highly localized diversity over spatial scales as small as several kilometers. We hypothesize that population structure patterns on the FRT may be a result of frequent sexual reproduction in the past which has been tempered during recent population declines and given way to a series of dynamically interconnected populations which now rely primarily on fragmentation for reproduction. Genetic diversity produced in this way is also likely supplemented by somatic mutations (Van Oppen, Souter, Howells, Heyward, & Berkelmans, 2011) which are propagated asexually in long‐lived individuals (Devlin‐Durante, Miller, Precht, & Baums, 2016). This concept reconciles the extremely rare discovery of definitively sexual recruits (Tunnicliffe, 1981) with the continued presence of diverse assemblages on individual reefs. Localized areas of variability in genetic diversity produce a more complex genetic landscape.

Notably, lower genetic diversity occurs in two areas: offshore of Miami Beach in Miami‐Dade and in the Upper Keys. Depressed local diversity in these areas may be related to differential mortality during long‐term Caribbean reef decline or localized stressors such as White Band Disease, bleaching, and storm impacts. We also hypothesize that the northern area of low diversity near Miami beach is due to the transition between reef habitat types (Banks et al., 2008) or influence of the Biscayne Bay “safety valve” tidal flow (Glynn, Szmant, Corcoran, & Cofer‐Shabica, 1989; Shinn, 1988; Wang & van de Kreeke, 1986). Interestingly, high diversity is found in nearby areas of Broward county, which also support dense thickets (large populations of living staghorn colonies with interlocking skeletons) that remain infrequent elsewhere on the FRT (Vargas‐Angel, Thomas, & Hoke, 2003; Walker, Larson, Moulding, & Gilliam, 2012). Based on the potential influence of inimical water quality on connectivity, we expected to see lower diversity in the Middle Keys where the influence of Florida Bay is strongest (Ginsburg & Shinn, 1995) and reef structure is most sparse (Ginsburg, Gischler, & Kiene, 2001), but found evidence of high diversity in this area and high population structure across this region. Resolving the influence of environmental heterogeneity on genetic diversity is an important area of further study, with valuable information for restoration and reef health.

Genetic patterns of Caribbean *A. cervicornis* exhibit extensive population structure and high variation in genetic diversity, even over small spatial scales. Sampling strategies used by management groups capture this variation and are capable of replicating the assemblages seen on wild reefs, but the resources and obstacles in each region make local management imperative. Setting goals to increase diversity (Miami‐Dade and Upper Keys) and protect unique or highly diverse areas (Lower Keys, Middle Keys, and Broward) may allow for more efficient and effective conservation of an important foundation species, with cascading effects on individual reefs and eventually throughout the species range.

## AUTHOR CONTRIBUTIONS

CD and DL designed and coordinated the study. CD completed library preparation and analysis and wrote the manuscript. SS, EG, EB, KM, KN, CM, MJ, and VG managed nursery resources, contributed samples, and contributed to manuscript review.

## DATA ACCESSIBILITY

The *Acropora cervicornis* unfiltered VCF and sampling locations are available on Dryad.
