# Supplementary material for: Genomic patterns in Acropora cervicornis show extensive population structure and variable genetic diversity
Source: Ecol Evol. 2017 Jun 30;7(16):6188–200. doi: 10.1002/ece3.3184 (PMC5574808; doi:10.1002/ece3.3184)
Supplement: Supplementary file 1 [file ECE3-7-6188-s001.pdf]

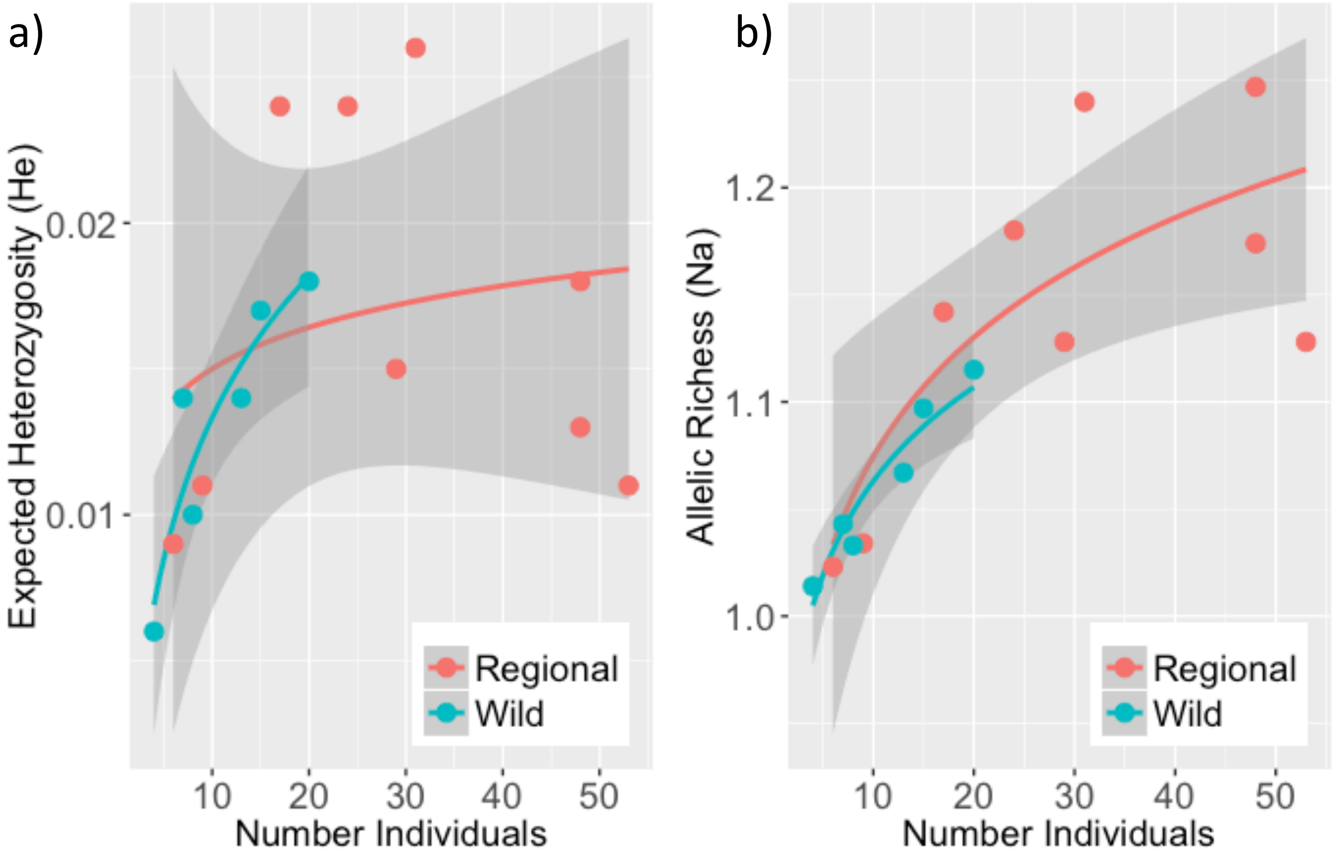

Figure S1 – Change in a) Allelic Richness (Na) and b) Expected Heterozygosity (He) with sample size.

a) Allelic Richness (Na) for wild and regional populations, comparing sample sizes and logarithmic growth of Na.

b) b) Expected Heterozygosity (He) for wild and regional populations. Red symbols represent wild collections, blue symbols represent regional collections.

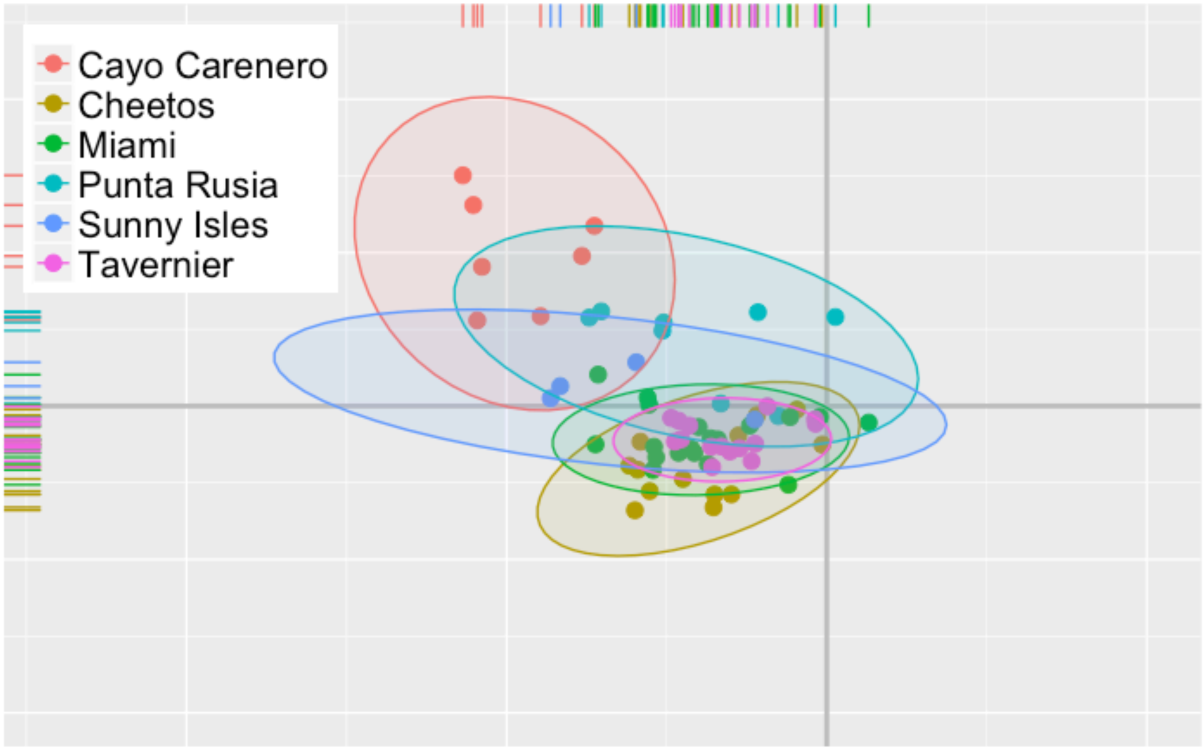

Figure S2 – DAPC for wild populations
Discriminant Analysis of Principal Components for Wild populations in Florida (n=4) and the Dominican Republic (n=2).

	Cayo Carenero	Punta Ruisa	Cheetos	Miami-1	Sunny Isles	Tavernier
**Cayo Carenero**		0.142	0.145	0.134	0.062	0.138
**Punta Rusia**	0.010		0.168	0.121	0.129	0.124
**Cheetos**	0.001	0.000		0.080	0.099	0.053
**Miami**	0.000	0.000	0.001		0.015	0.062
**Sunny Isles**	0.794	0.119	0.266	0.823		0.039
**Tavernier**	0.001	0.003	0.125	0.008	0.720	

Table S1 – Pairwise F_{ST} values between populations.
Pairwise F_{ST} values calculated by Arlequin (3.5.2) above the diagonal (darker colors = higher values) with p-values calculated from 10,000 permutations. Red shading represents significant comparisons at after Benjamini-Hochberg procedure with a 10% FDR.
